# Novel *Thermus thermophilus* and *Bacillus subtilis* mixed‐culture ferment extract provides potent skin benefits in vitro and protects skin from aging

**DOI:** 10.1111/jocd.16531

**Published:** 2024-08-19

**Authors:** Jingyi Wang, Hu Huang, Kan Tao, Lili Guo, Xincheng Hu, Huailong Chang

**Affiliations:** ^1^ Global R&D Center Shanghai Chicmax Cosmetic Co. Ltd. Global Harbor Tower B Shanghai China

**Keywords:** anti‐aging, anti‐inflammation, anti‐oxidation, autophagy, mixed‐culture fermentation

## Abstract

**Background:**

Skin aging is one of the most abundant aging‐related disorders that can be accelerated by excessive exposure to ultraviolet irradiation. Topically applied fermented skincare ingredients have gained mounting attentions due to their high concentration of various skin nourishing nutrients and bioactive components and low skin irritation potency.

**Aims:**

In the present study, we aim to fully demonstrate the skin‐related benefits of a novel extract of *Thermus thermophilus* and *Bacillus subtilis* mixed‐culture ferment (TBFE).

**Methods:**

TBFE was prepared through an innovative mixed‐culture fermentation process. The contents of nutrients and bioactive ingredients were quantified by different methods accordingly. Both in vitro tests and randomized controlled human trial were utilized to further demonstrate multifaceted beneficial effects on human skin, as well as the potential mechanisms.

**Results:**

Our results showed that TBFE upregulated the expression of type IV collagen, elastin, aquaporin‐3, and dermal‐epidermal junction markers, while inhibited production of melanin, in different skin cell models. Moreover, TBFE inhibited the generation of reactive oxygen species and pro‐inflammatory mediators induced by ultraviolet irradiation in normal human keratinocytes, while stimulated autophagy in senescent keratinocytes. Results from clinical studies confirmed those in vitro findings, demonstrating that TBFE at 5% and 20% concentration provides anti‐aging properties in subjects with sensitive skin, in terms of improving wrinkles, moisturization, and skin lightening.

**Conclusions:**

In summary, we demonstrate that a novel mixed‐culture ferment extract has promising anti‐aging effects, which may be attributed to anti‐oxidation, anti‐inflammation, and promotion of autophagy in skin cells.

## 1 INTRODUCTION

Skin aging is caused by intrinsic (chronological aging) and extrinsic factors (environmental aging), including sun radiation, air pollution, lack of sleep, and lifestyle.^1^ The environmental stressors account for about 90% of accelerated aging, causing skin to be dehydrated and less elastic, ultimately resulting in the appearance of fine lines and wrinkles.^2^ Aged skin is also characterized by a thinner epidermis, a flattened dermal‐epidermal junction (DEJ), and degradation of the extracellular matrix (ECM), especially collagen and elastin fibers.^3^ DEJ is a complex network of interconnecting proteins with finger‐like projections of rete ridges that help to maintain the connection between the epidermis and dermis. A flattened DEJ caused by accelerated aging results in less surface area, increased skin fragility and reduced supply of nutrients and oxygen.^2^ Moreover, the main components of anchoring plaques and fibrils in the DEJ, such as Types IV and VII collagen, decrease with age.^4^ The accumulation of senescent epidermal keratinocytes and dermal fibroblasts has also been correlated to skin aging. Chronic exposure to extrinsic factors causes oxidative stress, DNA damage, and dysfunctional macromolecules leading to cell senescence.^5^ Senescent cells lose their capacity to proliferate and adopt a senescence‐associated secretory phenotype (SASP), resulting in the secretion of senescence‐inducing factors, including several pro‐inflammatory cytokines that affect neighboring cells.^6^ In addition, autophagy, which eliminates cell senescence inducers, is suppressed in senescent cells. Therefore, to help slow skin aging and mitigate its deleterious effects, it would be advantageous for topical ingredients and formulations to alleviate inflammation, stimulate the production of ECM components, strengthen the DEJ adhesion of epidermis and dermis, and promote autophagy of senescent cells.

Cosmetic products containing natural actives, including fermented extracts, are gaining popularity among consumers. *Thermus thermophilus* (*T. thermophilus*) was originally isolated from a thermal vent within a hot spring in Japan.^7^ As a thermophilic bacterium, *T. thermophilus* has many extremophilic proteins that remain active and structurally stable at high temperatures (>80°C).^8^ Extracts of *T. thermophilus* are rich in polyamines such as spermidine and spermine because they are essential for protein synthesis at high temperatures.^9^ It has been hypothesized that polyamines can elongate the life span of many species, possibly through stimulating autophagy.^10^ Natto is a traditional, healthy Japanese food of soybeans fermented by *Bacillus subtilis* (*B. subtilis*). Fermented soybeans are also rich in polyamines and spermidine, which can be increased during fermentation along with the overall peptide content.^11^ Additionally, the fermentation of soybeans produces a strong fibrinolytic enzyme named nattokinase, which is widely used as an oral agent for thrombolytic therapy.^12^ Hence, fermentation could not only retain effective components in substrates but also increase existing and produce new bioactive or bioavailable ingredients through decomposition and transformation. However, no study is known to have explored the combined effects of fermentation by the two microorganisms under a mixed‐culture condition.

Here, we introduce for the first time a novel *T. thermophilus* and *B. subtilis* ferment ex‐tract (TBFE) obtained from a mixed‐culture fermentation. Potential anti‐aging benefits of the fermented extract were evaluated through in vitro cell models and randomized controlled human studies.

## 2 MATERIALS AND METHODS

### 2.1 Preparation of *T. thermophilus* and *B. subtilis* Ferment Extract

TBFE was prepared by a multi‐step and mixed‐culture fermentation procedure. Two basal media were prepared for the culture of *T. thermophilus* and *B. subtilis*, respectively. For *T. thermophilus*, the medium comprised tryptone (10 g/L), yeast extract (10 g/L), (NH_4_)_2_SO4 (10 g/L), MgSO4 (2 mM), KH_2_PO4 (4 mM), CaCl_2_ (0.3 mM), FeCl_3_ (0.5 mM), ZnSO4 (0.3 mM), CuSO4 (0.1 mM), and Na_2_MoO4 (0.1 mM). For *B. subtilis*, the medium comprised chickpea floor (10 g/L), tryptone (15 g/L), beef extract (10 g/L), NaCl (5 g/L) and (NH_4_)_2_SO4 (5 g/L). *T. thermophilus* (BAA‐163, ATCC) was precultured overnight at 70°C in a medium containing tryptone (8 g/L), yeast extract (4 g/L), and NaCl (3 g/L) as the seed culture, while *B. subtilis* (CICC10023, CICC) was cultured overnight at 37°C in LB medium. For flask cultures, 500 mL flasks containing 100 mL of the basal media were inoculated with 1 mL of the seed culture and cultured at the specified temperatures (65°C for *T. thermophilus*, 30°C for *B. subtilis*) for 12 h with 200 rpm shaking. Batch fermentation was carried out in a 5 L stirred tank bioreactor containing 2 L basal medium for *T. thermophilus*. During the first step of fermentation, a 5% (v/v) inoculum of *T. thermophilus* from flask culture was inoculated to the medium and the fermentation was performed at 65°C for 16 h with an aeration rate of 1.0 vvm (air volume/medium volume/minute). Then, the system was cooled down to 30°C and a 5% (v/v) inoculum of *B. subtilis* was added. Fermentation for the second step was performed at 30°C for additional 16 h with an aeration rate of 1.5 vvm. The pH was maintained at 7.5 by adding 3 M NaOH through the batch fermentation and the agitation rate was controlled between 100 and 650 rpm to maintain a dissolved oxygen of 30 ± 5%. Upon ending of the fermentation, the resulting broth was centrifuged to collect the supernatant, which was then decolorized with 0.3% (m/v) baked active carbon, filtered, and centrifuged again to obtain a clear, pale‐yellow solution. The solution was treated with preservatives (p‐hydroxyacetophenone and 1,2‐hexanediol) and then filtered to remove microorganisms to obtain the final TBFE product.

### 2.2 Quantification of bioactive contents in the TBFE

The content of total protein was measured by Coomassie brilliant blue G‐250‐binding approach, with the optical density recorded at 595 nm and compared to a standard curve. Content of total peptide was measured by Biuret test, and the range of peptide concentration was determined by colorimetry method. Content of polyphenol was determined by Folin–Ciocalteu method, which was qualified by the amount of phosphomolybdate and phosphotungstate reduced by polyphenols. Content of polyamine was measured by derivatization with benzoyl chloride and then tested by high‐performance liquid chromatography (HPLC). Content of glutathione (GSH) was measured using commercial GSH test kit (Beyotime, Shanghai, China) by reacting with DTNB (5,5′‐dithiobis‐2‐nitrobenzoic acid) to get a yellow product, which could be qualified spectrophotometrically at 412 nm. Con‐tent of nattokinase was determined by hydrolyzing the peptide bond of fibrin, which resulted in changes of optical density at 275 nm. Fibrin Units (FU) refers to the amount of enzyme consumed when the optical density increases by 0.01 at 275 nm. Non‐volatile residue (NVR) was measured by weighting the residue after evaporation of the solvent.

### 2.3 Cell culture and treatment

We adopted various skin originated cell lines to explore the potential effects of TBFE on human skin in vitro. Primary human dermal fibroblasts (HDFs) (Biocell Biotechnology, Guangdong, China), which were seeded in Dulbecco's Modified Eagle's Medium (DMEM) supplemented with 10% fetal bovine serum (FBS). Cells were treated with test materials for 24 h before harvesting. Primary human epidermal keratinocytes (NHEKs) were purchased from Thermo‐Fisher (MA, USA), and were seeded in EpiLife™ medium with hu‐man keratinocyte growth supplement (Thermo‐Fisher, MA, USA) and incubated for 24 h. NHEKs were also treated with test materials for 24 h.

### 2.4 RNA extraction and polymerase chain reaction

Harvested cells were washed with PBS and total RNA was extracted using RNAiso Plus (TaKaRa, Beijing, China). PrimeScriptTM RT reagent kit (TaKaRa, Beijing, China) was used for the first‐strand cDNA synthesis. For the quantification of type IV collagen (*COL4A4*) and elastin (*ELN*), quantitative RT‐PCR was performed using STBR Green Real Time PCR Master Mix (TaKaRa, Beijing, China) in a CFX96 detection system (Bio‐Rad, CA, USA). The primers used were as follows: *COL4A4* forward: 5′‐CAGGCTCAACTGGTCTAAGAGG‐3′, reverse: 5′AGGTGGACCAAAGTGACTGGCA‐3′; *ELN* forward: 5′‐TCCAGGTGTAGGTGGAGCTT‐3′, reverse: 5′GTGTAGGGCAGTCCATAGCC‐3′. For the quantification of occludin (*OCLN*), syndecan 1 (*SDC1*), perlecan (*HSPG2*), and chondroitin sulfate proteoglycan 8 (*CD44*), quantitative RT‐PCR was performed using TaqMan® Fast Advanced Master Mix (Life Technologies, MA, USA) and TaqMan® Gene Expression Assays (Life Technologies, MA, USA) with specific Taqman® probes for *OCLN* (Hs01049883_m1), *SDC1* (Hs00174579_m1), *HSPG2* (Hs00194179_m1), and *CD44* (Hs01075862_m1). Gene expression was calculated via 2−^ΔΔCt^ method using GAPDH as an internal control.

### 2.5 Aquaporin‐3 assay

Human epidermal keratinocyte cell lines (HaCaTs) were seeded in RPMI‐1640 medium (Thermo‐Fisher, MA, USA) supplemented with 10% FBS and incubated for 24 h. Cells were treated with fresh media containing test materials and incubated for 48 h. Membrane proteins were collected using Mem‐PER™ Plus Kit (Thermo Fisher, MA, USA) and AQP3 was measured by enzyme‐linked immunosorbent assay following manufacturer's instructions (MyBioSource, CA, USA).

### 2.6 Melanin content assay

Normal human melanocytes (NHMCs) were seeded in M254 medium (Biocell Bio‐technology Co., Ltd. Guangdong, China) with 10% FBS (Royacel, Gansu, China) and incubated for 24 h. Cells were treated with test materials for 72 h, with fresh treatment media being administered every 24 h. After treatments, cells were washed with PBS and harvested with 0.25% trypsin for 1 minute, and the supernatant was dis‐carded after centrifugation. The lysate was extracted with a mixed solution of water, ethanol, and diethyl ether (2:5:5, v/v) for 30 min, centrifuged and lysed with 1 M NaOH for 40 min at 80°C. Supernatants were measured by monitoring the optical density at 405 nm and compared to a synthetic melanin standard curve.

### 2.7 ROS and IL‐6 detection in a UVB irradiation model

NHEKs were seeded in DMEM with 10% FBS and incubated overnight. Cells were pre‐treated with test materials for 24 h, then washed with PBS and irradiated with 300 mJ/cm^2^ UVB. Cells were then incubated without test materials for an additional 24 h. For IL‐6 cytokine assay, culture media supernatants were collected and measured by ELISA (Abcam, MA, USA). For ROS assay, cells were washed and incubated with DCFH‐DA (2′,7′‐Dichlorofluorescein diacetate) redox probe (Beyotime, Shanghai, China) for 30 min. Culture media was then discarded and cells were washed and harvested with trypsin. Resulting fluorescence intensity was measured by flow cytometry.

### 2.8 LC3‐II detection

BlumilightTM (Ashland, NC, USA) is an extract of Theobroma cacao seed and was used as positive control in the autophagy assay. HaCaTs were seeded in DMEM supplemented with 10% FBS and incubated for 24 h. Earle's Balanced Salt Solution (EBSS) was used as nutrient‐deprived media, and the full‐nutrient control group was cultured in 10% FBS‐supplemented DMEM. Treatments were diluted in EBSS and incubated for 4 h. Then, treatment media was replaced with cytosolic pro‐microtubule‐associated protein 1 light chain 3 (LC3) and LC3‐I removal reagents in PBS containing MgCl_2_ and CaCl_2_ and incubated for 5 min. Cells were washed three times with 2 mL PBS containing MgCl_2_ and CaCl_2_ and incubated with radioimmunoprecipitation assay (RIPA) buffer (Cell Biolabs, San Diego, CA, USA) for 10 min on ice. Cells were then detached and centrifuged to collect the supernatant, and the total protein concentration was deter‐mined by bicinchoninic acid (BCA) assay. The lysate was used to measure LC3‐II by ELI‐SA kit from Cell Biolabs (San Diego, CA, USA).

### 2.9 Human studies

To further prove the skin protecting effects of TBFE in human subjects, two separate clinical studies were conducted by a third‐party research organization to evaluate the effects of two lotions with different concentrations of TBFE. A 5% concentration was first chosen as a bench control corresponding to the in vitro dosing, while a 20% concentration was chosen as an exploratory concentration so that we may assess the dose–response relationship between TBFE and its effects. The 20% concentration was selected based on our safety test results, which demonstrated that only 2/30 subjects had faint erythema (doubtful reaction) after 24 h of exposure to a 20% TBFE solution (data not shown). The studies followed the guidelines of the Declaration of Helsinki and were approved by the ethic committee boards of the institute (Approval No. SHCPCH210607090). The following inclusion criteria were used: healthy adults aged between 18 and 60; sensitive skin that confirmed by lactic acid sting test; and wrinkles in the eye or facial area with scores ranged from 3 to 6 according to the SGS standard scales assessed by experienced dermatologists. And subjects with the following conditions were excluded from studies: pregnant or breastfeeding women; subjects with obvious skin lesions or scars on the test area; current participants in other human studies; and other reasons deemed by the institute to be ineligible for the study. All 30 subjects of both studies received the lotion and were instructed to apply it twice daily after facial cleansing for 28 days. Data for skin tone, barrier function, and skin hydration were collected at base‐line (Day 0) and every 7 days thereafter. Skin tone ITA° was measured by Colorimeter® CL400, barrier function via TEWL by Tewameter® TM300, and skin hydration by Corneometer® CM825 (Courage+Khazaka electronic GmbH, Köln, Germany). Self‐perception questionnaires (SPQs) and digital photos were also collected at each timepoint. Photography and skin wrinkle area analysis were performed with VISIA®‐CR and PRIMOS‐CR (Canfield Scientific, Parsippany, NJ).

### 2.10 Statistical Analysis

Statistical significance for in vitro assays was determined by ANOVA followed by a Dunnett multiple‐comparisons test using *p* < 0.05 as a significant difference. For human studies data, statistical significance was determined by a two‐tailed *t*‐test with *p* < 0.05 considered to be significant difference.

## 3 RESULTS

### 3.1 The nutrients and bioactive components contained in TBFE

The final yield of TBFE was a yellowish transparent fluid with characteristic smell. We first identified and quantified characteristic components that are known to have skin improving effects. As shown in Table 1, TBFE contained a variety of active ingredients, including proteins, peptides, polyphenols, polyamines, glutathione, and nattokinase. The most abundant ingredients belonged to the polyamine family (3.1 g/L), followed by pep‐tides (0.15–0.3 g/L) and polyphenols (21.01 mg/L). Glutathione was determined to be 24.76 μmol/L. Importantly, nattokinase, the special active ingredient derived from the fermentation by *B. subtilis*, was quantified to be 18.72 FU/mL.

**TABLE 1:** The composition of nutrients and active ingredients in TBFE.

Components	Concentration
Protein	13.78 mg/L
Peptide	0.15–0.3 g/L
Polyphenol	21.01 mg/L
Polyamine	3.1 g/L
Glutathione	24.76 μmol/L
Nattokinase	18.72 FU/mL
Non‐volatile residue	0.33%

### 3.2 TBFE demonstrated potent skin improving properties in vitro

**FIGURE 1:**
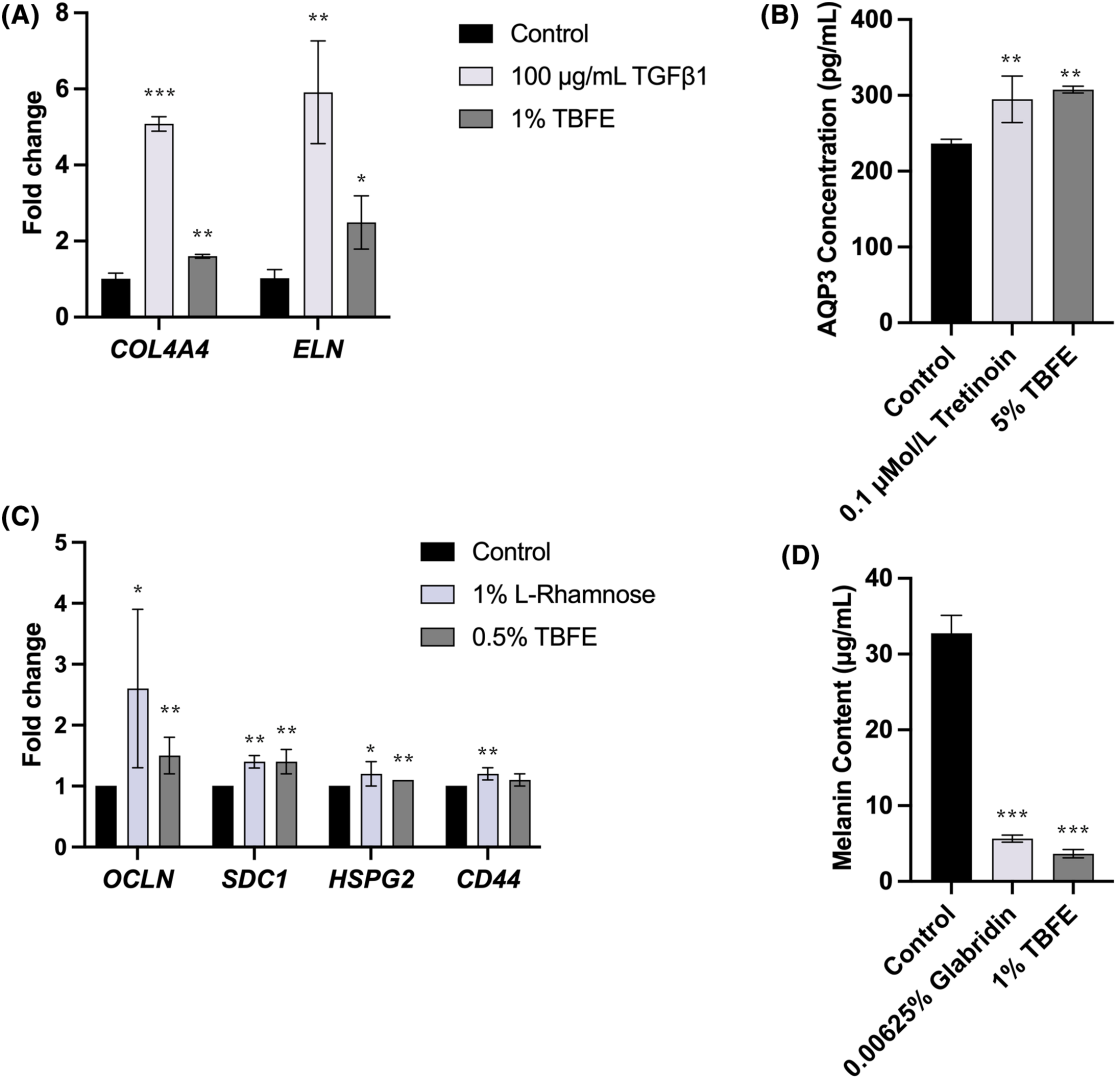
(A) TBFE (1%, v/v) upregulated gene expression of type IV collagen (*COL4A4*) and elastin (*ELN*) in HDFs after 24‐hour treatments. (B) TBFE (0.5%, v/v) upregulated gene expression of *OCLN*, *SDC1*, and *HSPG2* in NHEKs after 24‐hour treatments. (C) TBFE (5%, v/v) improved AQP3 content in HaCaTs. (D) TBFE (1%, v/v) reduced melanin content in NHMCs. Data represents the mean ± SD (*n* = 3). *p < 0.05, **p ≤ 0.01, and ***p ≤ 0.001 indicate a statistically significant difference relative to control cells.

In the current study, we first employed multiple skin cell models to explore the potential skin benefits that may be brought by TBFE. Figure 1A illustrated the impacts of TBFE treatment on the transcriptional levels of Type IV collagen and elastin in primary human dermal fibroblasts (HDFs), of which the expression levels have been well recognized as key skin aging markers. Gene expression of type IV collagen and elastin after 24 h treatment with 1% TBFE increased significantly by 60% and 49%, respectively, which was comparative to the effects of transforming growth factor beta (TGF‐β). In addition, the effects of TBFE on gene expression of key regulators in maintaining the integrity of DEJ was also explored. In primary human epidermal keratinocytes (NHEKs) treated with 0.5% TBFE, the expression of *OCLN*, *SDC1*, and *HSPG2* were significantly upregulated by 54%, 42%, and 14%, respectively (Figure 1B). As a pathway for water to exit, aquaporin 3 (AQP3) plays key roles in skin hydration and water retention. In the current study, treatment with 5% TBFE significantly increased the synthesis of AQP3 in human epidermal keratinocyte cell lines (HaCaTs) by 30% compared to in untreated cells (Figure 1C). We further investigated the potential effects of TBFE on skin brightening by evaluating melanin production in human melanocytes. The results showed that treatment with 1% TBFE significantly inhibited the melanin production by 89% in human melanocytes (Figure 1D). Together, our data indicates that TBFE may provide various skin benefits, in terms of promoting collagen and elastin expression, DEJ integrity, and water retention, while inhibiting melanin production.

### 3.3 TBFE protected skin from aging in human subjects

Based on the multiple skin benefitting properties demonstrated by TBFE in vitro, we next sought to determine if TBFE could provide actual benefits in human subjects. Two separate clinical studies were conducted to evaluate the effects of two lotions containing either 5% (Lotion A) or 20% (Lotion B) TBFE as the only active ingredient. The study for 5% TBFE lotion enrolled 30 Asian adults (25 females and 5 males) with sensitive skin ranging in age from 34 to 60 years of age (average age of 50.3 ± 8.2). The study for 20% TBFE lotion also enrolled 30 Asian adults (26 females and 4 males) with sensitive skin ranging in age from 38 to 59 years of age (average of 49.2 ± 6.3).

Results of all parameters measured by instruments can be found in Table 2. Skin tone individual typology angle (ITA°) showed a significant increase by 5% for lotion A and 8% for lotion B at the end of the study compared to baseline. At Day 7 and day 28, skin tone values for lotion B were also significantly higher compared to lotion A, suggesting TBFE exerted its effects in a dose‐dependent manner. In the meantime, the trans‐epidermal water loss (TEWL) of skin at the test area significantly decreased compared to baseline after 7 and 28 days of application for both lotions A and B (by 9% and 20% for lotion A, 11% and 23% for lotion B, respectively). In consistent with reduction in TEWL, the application of both lotion A and lotion B significantly increased stratum corneum (SC) hydration after 7 and 28 days.

**TABLE 2:** Summary of instrument measured endpoints for lotions containing TBFE.

Endpoint (Mean ± SD)	Lotion A (5% TBFE)			Lotion B (20% TBFE)		
	Day 0	Day 7	Day 28	Day 0	Day 7	Day 28
skin tone ITA^a^	39.44 ± 7.70	40.02 ± 7.45*	41.42 ± 7.55**	38.90 ± 7.62	40.40 ± 7.93*,^#^	42.01 ± 7.64**,^#^
TEWL^b^	17.40 ± 2.60	15.84 ± 2.79**	13.90 ± 3.49**	17.58 ± 3.02	15.66 ± 3.31**	13.51 ± 3.17**
SC hydration^a^	44.51 ± 10.97	49.33 ± 11.07**	56.56 ± 9.63**	42.72 ± 10.30	49.60 ± 10.36**	57.77 ± 8.67**

Abbreviations: SC, stratum corneum; TEWL, trans‐epidermal water loss.

* *p* < 0.05 and **p ≤ 0.01 compared to values of Day 0 (baseline).

^#^ *p* < 0.05 compared lotion A with lotion B.

^a^ A larger mean value indicates a better skin condition.

^b^ A smaller mean value indicates a better skin condition.

**FIGURE 2:**
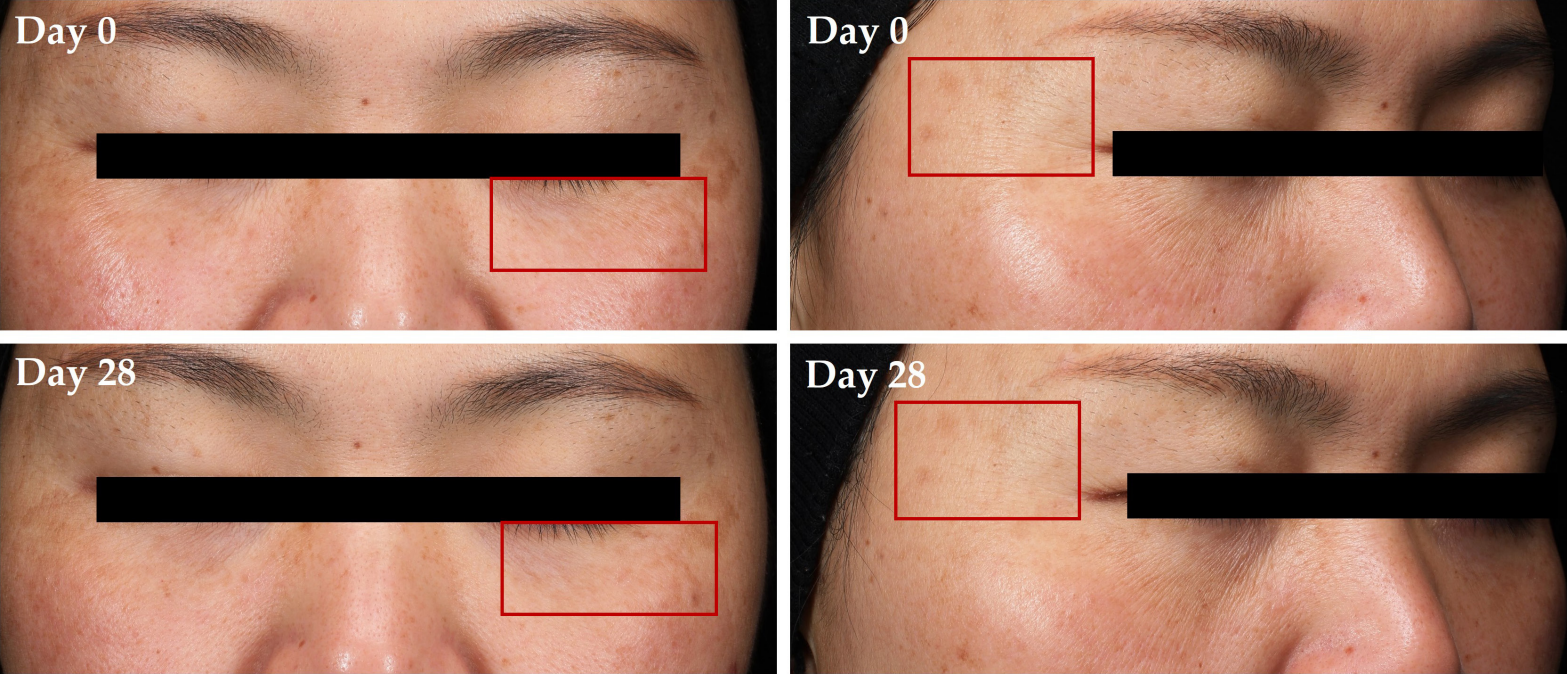
TBFE improved skin aging in human subjects. Standardized photographs of the face were taken at baseline (Day 0) and Day 28 after application of lotion B containing 20% TBFE. Skin wrinkles were analyzed using a VISIA‐CR Imaging system. Red boxes indicate the areas of visible improvements in skin wrinkles.

Dermatologist evaluation using SGS (Société Générale de Surveillance) standard scales, where a smaller value means a better skin condition, showed a significant decrease in periorbital lines, forehead lines, and facial wrinkles for both lotions A and B (Table 3). Moreover, there was a significant improvement for periorbital lines and forehead lines for both lotions after 28 days (15% and 18% for lotion A, 20% and 22% for lotion B, respectively). After 7 days of application, the value for facial wrinkles of lotion B was significantly better than of lotion A. There was also a significant improvement from baseline by nearly 12% for skin elasticity and tightening for both lotions after 28 days. Images captured at baseline and day 28 further demonstrated the improvement in skin wrinkles (Figure 2). In consistent with instrument measurement and dermatologist evaluation, the self‐perception of the subjects also showed that more than 90% of subjects were satisfied with the effects of both lotions on skin condition improvements, including facial wrinkles, sensitivity, hydration, and firmness (Data not shown).

**TABLE 3:** Summary of skin condition evaluated by dermatologists for lotions containing TBFE.

Endpoint^a^ (Mean ± SD)	Lotion A (5% TBFE)			Lotion B (20% TBFE)		
	Day 0	Day 7	Day 28	Day 0	Day 7	Day 28
Periorbital lines	4.57 ± 0.84	4.30 ± 0.87**	3.88 ± 1.04**	3.95 ± 0.79	3.65 ± 0.78**	3.15 ± 0.90**
Forehead lines	4.47 ± 0.91	4.17 ± 0.91**	3.67 ± 1.09**	3.87 ± 0.81	3.53 ± 0.83**	3.02 ± 0.85**
Facial wrinkles	4.52 ± 0.77	4.33 ± 0.84**	3.88 ± 0.90**	4.32 ± 0.76	3.97 ± 0.81**,^#^	3.55 ± 0.79**
Skin elasticity	4.72 ± 0.74	4.60 ± 0.77**	4.17 ± 0.79**	4.50 ± 0.60	4.27 ± 0.63**	3.92 ± 0.46**
Skin tightening	4.72 ± 0.74	4.60 ± 0.77**	4.17 ± 0.79**	4.50 ± 0.60	4.27 ± 0.63**	3.92 ± 0.46**

*Note*: **p ≤ 0.01 compared to values of Day 0 (baseline).

^#^ *p* < 0.05 compared lotion A with lotion B.

^a^ A smaller mean value indicates a better skin condition.

### 3.4 TBFE may act to protect skin by ROS scavenging, inflammation alleviation, and autophagy promotion

**FIGURE 3:**
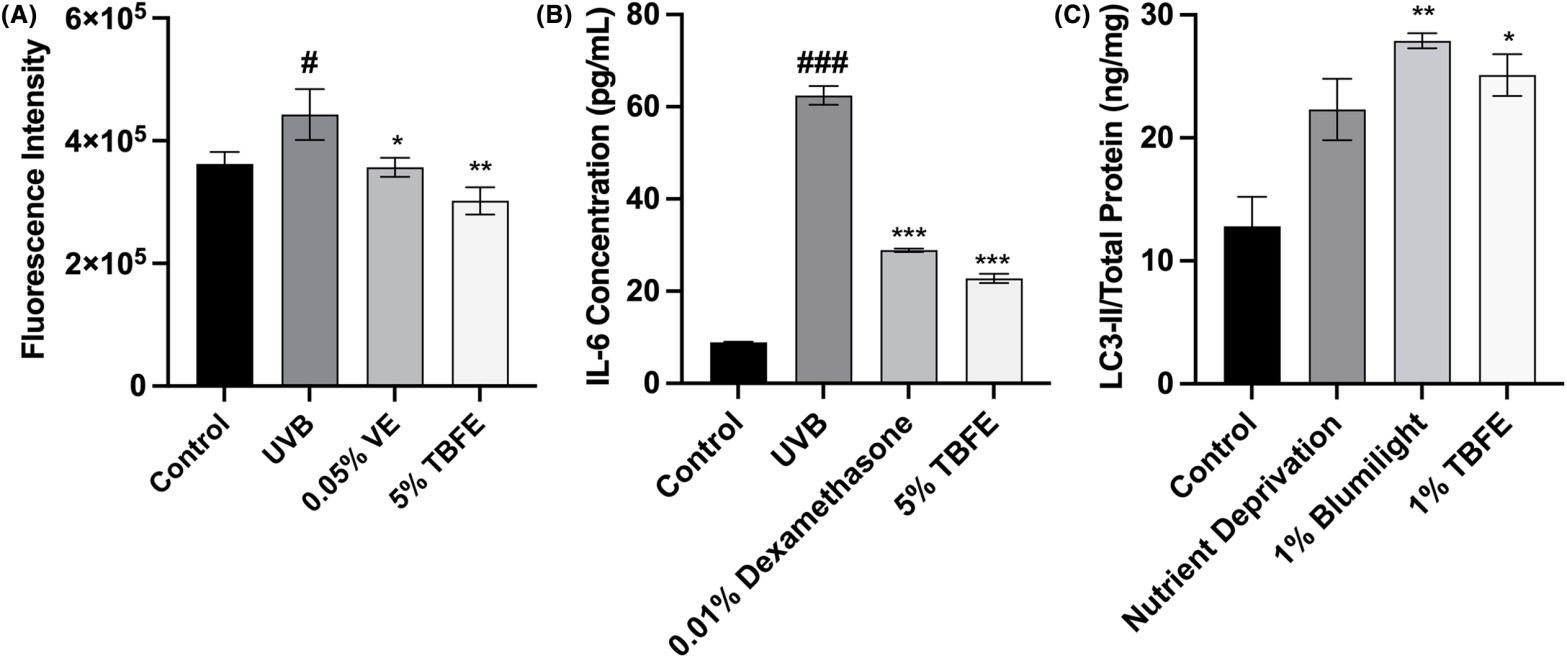
TBFE (5%, v/v) suppressed UVB (300 mJ/cm^2^) induced ROS (A) and IL‐6 (B) production in NHEKs. (C) TBFE (1%, v/v) upregulated the relative content of LC3‐II in HaCaTs. Vitamin E (0.05%), Dexamethasone (0.01%), and BlumilightTM (1%, v/v) were used as positive controls in the corre‐sponding situations. Data were presented as mean ± SD (*n* = 3). *p ≤ 0.05, **p ≤ 0.01, and ***p ≤ 0.001 indicate a statistically significant difference relative to untreated control cells. ^#^ *p* ≤ 0.05 and ^###^ *p* ≤ 0.001 indicates significant difference relative to UVB‐only cells.

Given the various effects of TBFE demonstrated and the fact that it is a mixture of nutrients and bioactive agents, we hypothesized that TBFE may exert its effects by different mechanisms. Thus, we first established a UVB irradiation model to explore the potential mechanisms, in terms of anti‐oxidation and anti‐inflammation. UVB irradiation significantly induced reactive oxygen species (ROS) generation by 22% in NHEKs (Figure 3A). Treatment with 5% TBFE reduced ROS by 32% compared to UVB‐only treated cells. Interestingly, TBFE even reduced ROS generation by 17% compared to untreated cells. UVB irradiation also significantly induced pro‐inflammatory cytokine production of interleukin 6 (IL‐6), while treatment with 5% TBFE significantly inhibited IL‐6 production by 64% in NHEKs (Figure 3B). Lastly, the potential involvement of autophagy was also explored by detecting the effect of TBFE on the microtube associated protein 1 light chain 3 (LC3‐II) content in HaCaTs. Nutrient deprivation alone, while not significant, increased LC3‐II content to 22.3 ng/mg compared to untreated, full nutrient control (12.8 ng/mg). Importantly, treatment with 1% TBFE significantly increased LC3‐II levels to 25.1 ng/mg compared to full nutrient control, which was an additional 13% increase over the effect of the nutrient deprivation group (Figure 3C).

## 4 DISCUSSION

In the present study, a mixed‐culture fermentation by *T. thermophilus* and *B. subtilis* was conducted, from which a ferment extract was prepared to explore its potential effects on skin's well‐being (Supplementary Table S1). Our TBFE consists of multiple skin nourishing nutrients and bioactive components, of which polyphenols are the mostly recognized for their antioxidant and/or anti‐inflammation properties. Those effects are primarily attributed to their function in inhibiting the conversion of arachidonic acids to pro‐inflammatory prostaglandins, and/or inhibiting the activation of transcription factor nuclear factor‐κB (NF‐κB).^13^ Polyphenols have also been reported to activate autophagy through the activation of sirtuin 1 (SIRT1) protein, which further activates autophagy‐related proteins ATG5, ATG7, and LC3.^14^ In addition to polyphenols, TBFE also contains relatively a large number of polyamines, among which spermidine has been shown to extend the lifespan of yeasts, flies, worms, mice, and human immune cells through upregulating autophagy‐related transcripts.^10^, ^15^ Moreover, the glutathione (GSH) contained in the TBFE has also been involved in the nutrient metabolism and regulation of cellular events, including cytokine production and immune response.^16^ In addition, GSH is also commonly used in cosmetic formulations for skin‐lightening purposes, because it can scavenge free radicals and inhibit the activation of tyrosinase.^17^, ^18^ Importantly, nattokinase is the most special component identified in the TBFE, because it is newly produced by *B. subtilis* during fermentation. Recently, nattokinase has been suggested to suppress endothelial inflammation and increase autophagy in mice.^19^ Altogether, the rich contents of bioactive components and their functions thereof provide TBFE the possibility to act as an effective anti‐aging ingredient, and can in part explain its complex roles in anti‐oxidation, anti‐inflammation, and promoting cell autophagy.

Our data demonstrates the potential anti‐aging effects of TBFE by regulating the expression of various skin aging markers in vitro, including the type IV collagen and elastin, which are among the main contributors to the backbone of base membrane and skin elasticity, respectively.^20^, ^21^ Moreover, TBFE upregulated the gene expression of essential components for DEJ, including *OLCN*, *SDC1*, and *HSPG2*. Therefore, we hypothesize that TBFE has the potential to reduce facial wrinkles by enhancing the synthesis of ECM. In addition, AQP3 is the most abundant aquaporin in the skin and helps to keep skin hydration, which also plays key role in prevent skin from aging.^22^ In the current study, TBFE significantly stimulated the production of AQP3 while suppressed the production of melanin, suggesting the possibility of TBFE to improve skin hydration and skin tone. Indeed, our human data confirmed all these potential benefits. After 4 weeks of application of lotions containing 5% or 20% TBFE, human subjects showed significant improvements in various skin parameters, such as skin tone, facial wrinkles, and skin elasticity. The scores for facial wrinkles decreased significantly as evaluated by dermatologist, and over 90% of human subjects reported satisfaction with the improvements in their skin condition. All these data explicitly indicates that TBFE prepared in the current study is an anti‐aging ingredient for topically applied skincare products.

To provide further evidence for the mechanisms of TBFE underling the improvement of skin conditions, we investigated its effects on ROS scavenging, inflammation alleviation, and cell autophagy. Chronic exposure to ultraviolet irradiation results in photo‐aging of the skin, represented by decreased elasticity, development of fine lines and wrinkles, increased roughness, and a disrupted skin barrier, which has been associated with increased ROS production and inflammation.^23^ On the other hand, autophagy is a metabolic process that maintains cellular homeostasis by scavenging cells with misfolded or aggregated proteins and damaged organelles, in response to nutrients deprivation and oxidative stress.^24^ Research has demonstrated the anti‐aging effects of autophagy on human skin via protecting the homeostasis of skin dermal fibroblasts, keratinocytes, and melanocytes against external and internal stresses.^25^ We demonstrate here TBFE alleviates the UVB induced damages by decreasing the contents of ROS and the inflammatory cytokine IL‐6. Moreover, LC3‐II, the marker of autophagy, was upregulated by TBFE, suggesting that TBFE may help to maintain cellular homeostasis via promoting metabolism and eliminating senescence inducers.

Though our current study has demonstrated promising results, there are still some limitations need to be addressed in the future. Firstly, the fermentation process for production of TBFE may be further optimized by responsive surface method. Secondly, the research on gene expressions was exclusively conducted on mNRA level. In‐depth research on the protein expression will provide more robust proof that TBFE can indeed alter the gene expression of key molecules in human skin. Regarding the clinical study, it was a single center study with relatively small number of subjects. Thus, the power of the clinical study may be further increased by including more subjects and expanding it to multi‐centers. Lastly, the study into the molecular mechanisms by TBFE to exert skin improving effects was relatively superficial. We will need more thorough research into the key signaling pathways that mediate its effects on cell autophagy, inflammation, and oxidation.

## 5 CONCLUSION

We conclude that a novel mixed‐culture ferment extract of *Thermus thermophilus* and *Bacillus subtilis* can exert anti‐aging effects both in vitro and in vivo, in terms of improving wrinkles, moisturization, and skin lightening, which may be attributed to its roles in anti‐oxidation, anti‐inflammation, and promotion of autophagy in skin cells.

## AUTHOR CONTRIBUTIONS

Conceptualization, K.T., H.H. and H.C.; methodology, J.W., K.T. and H.H.; investigation, J.W., K.T., L.G., and H.C.; data curation X.H., H.H. and H.C.; writing—original draft preparation, J.W. and H.C.; writing—review and editing X.H. and H.H.; visualization, J.W. and H.C.; supervision, H.H. and H.C.

## CONFLICT OF INTEREST STATEMENT

The authors declare no conflict of interest.

## ETHICS STATEMENT

The authors confirm that the ethical policies of the journal, as noted on the journal’s author guidelines page, have been adhered to and the appropriate ethical review committee approval has been received (Approval No. SHCPCH210607090). The guidelines of the Declaration of Helsinki were followed.

## Supporting information


Table S1.

## Data Availability

The data that support the findings of this study are available on request from the corresponding author. The data are not publicly available due to privacy or ethical restrictions.
