# Supplementary material for: Novel Thermus thermophilus and Bacillus subtilis mixed‐culture ferment extract provides potent skin benefits in vitro and protects skin from aging
Source: J Cosmet Dermatol. 2024 Aug 19;23(12):4334–42. doi: 10.1111/jocd.16531 (PMC11626318; doi:10.1111/jocd.16531)
Supplement: Supplementary file 1 — Table S1. [file JOCD-23--s001.docx]

***Table S1.*** *The key findings of the current study.*

| ***The study object*** | ***Testing models*** | ***Key findings*** |
| --- | --- | --- |
| *A novel Thermus thermophilus and Bacillus subtilis Mixed-Culture Ferment Extract (TBFE)* | *Primary human dermal fibroblasts (HDFs)* | *TBFE increased the expression of COL4A1 and ELN in HDFs.* |
|  | *Primary human epidermal keratinocytes (NHEKs)* | *TBFE increased the expression of OCLN, SDC1, and HSPG2 in NHEKs.* |
|  | *Human epidermal keratinocyte cell lines (HaCaTs)* | *TBFE increased the contents of AQP3 and autophagy marker LC3-II in HaCaTs.* |
|  | *Normal human melanocytes (NHMCs)* | *TBFE reduced the melanin content in NHMCs.* |
|  | *UVB irradiated NHEKs* | *TBFE reduced the production of cytokine IL-6 and ROS in NHEKs.* |
|  | *Human studies* | *TBFE improved skin tone, SC hydration, and skin elasticity, while decreased TEWL and wrinkles in human subjects.* |
